# Monkeypox Disease (MPOX) Perceptions among Healthcare Workers versus General Population during the First Month of the WHO Alert: Cross-Sectional Survey in Saudi Arabia

**DOI:** 10.3390/vaccines10122071

**Published:** 2022-12-03

**Authors:** Mohamad-Hani Temsah, Fadi Aljamaan, Shuliweeh Alenezi, Noura Abouammoh, Khalid Alhasan, Shereen A. Dasuqi, Ali Alhaboob, Mohammed A. Hamad, Rabih Halwani, Abdulkarim Alrabiaah, Sarah Alsubaie, Fatimah S. Alshahrani, Fahad AlZamil, Ziad A. Memish, Mazin Barry, Jaffar A. Al-Tawfiq

**Affiliations:** 1 College of Medicine, King Saud University, Riyadh 11362, Saudi Arabia; 2 Pediatric Department, King Saud University Medical City, King Saud University, Riyadh 11362, Saudi Arabia; 3 Critical Care Department, King Saud University Medical City, King Saud University, Riyadh 11362, Saudi Arabia; 4 Department of Psychiatry, College of Medicine, King Saud University Medical City, King Saud University, Riyadh 11362, Saudi Arabia; 5 Department of Family Medicine, King Saud University Medical City, King Saud University, Riyadh 11362, Saudi Arabia; 6 Department of Pharmacy, King Khalid University Hospital, King Saud University Medical City, Riyadh 12372, Saudi Arabia; 7 Immunology Research Laboratory, College of Medicine, King Saud University, Riyadh 12372, Saudi Arabia; 8 Division of Infectious Diseases, Department of Internal Medicine, King Saud University Medical City, King Saud University, Riyadh 11362, Saudi Arabia; 9 Hubert Department of Global Health, Emory University, Atlanta, GA 30322, USA; 10 Infectious Disease Division, Department of Medicine, Indiana University School of Medicine, Indianapolis, IN 46202, USA; 11 Infectious Disease Division, Department of Medicine, Johns Hopkins University School of Medicine, Baltimore, MD 21218, USA

**Keywords:** monkeypox knowledge, MPOX, public versus HCW perceptions, emerging infectious disease global alert

## Abstract

**Background:** Monkeypox disease (MPOX) recently re-emerged in May 2022, causing international outbreaks in multiple non-endemic countries. This study demonstrates a novel comparison between the knowledge and perceptions of Saudi healthcare workers (HCWs) and the general public regarding MPOX. **Methods:** An online survey, conducted from 27 May to 5 June 2022, assessing participants’ MPOX and monkeypox virus (MPV) knowledge in terms of transmission, vaccination, isolation precautions, and their attitudes toward seeking more information. **Results:** A total of 1546 members of the public and 1130 HCWs completed the survey. Briefly, 61.3% of the public and 74.2% of HCWs showed interest in seeking more information about MPOX. Both groups had average overall mean MPOX knowledge scores. Members of the public holding university degrees and those showing high levels of worry regarding MPOX had significantly higher knowledge scores. However, HCWs showed a poor vaccination knowledge score, while only 57% recognized that MPOX can present similarly to COVID-19 in the early stages. Female HCWs and those with high self-rated MPOX awareness had significantly high knowledge scores. HCWs in secondary and tertiary centers had significantly higher knowledge scores. **Conclusion:** Both groups showed a decent attitude in terms of seeking more MPOX knowledge, which correlated positively with their worry about and awareness of the disease. These observations are mostly as a consequence of the ongoing COVID-19 pandemic, which encouraged the public and HCW to acquire more information about any novel emerging disease. Policymakers should make the most of this attitude in their awareness campaigns to prevent the spread of the disease and encourage vaccination in cases where it is needed. The knowledge gaps among HCWs were most evident in terms of clinical presentation and vaccinations; this problem needs addressing if we are to avoid further emerging MPOX cases.

## 1. Introduction

Monkeypox disease (MPOX) recently re-emerged in May 2022 and caused international outbreaks in multiple non-endemic countries [1,2]. As of 13 July 2022, a total of 10,845 laboratory-confirmed cases of MPOX were reported in 59 countries that had no historical report of the disease before that time [3]. Monkeypox virus (MPV) is a DNA virus that is a member of the genus *Orthopoxvirus* and is closely related to smallpox, a disease that caused more than 300 million fatalities during the twentieth century but was ultimately eradicated in 1980, thanks to global vaccination efforts led by the World Health Organization (WHO); this vaccination also has efficacy against MPV [4]. However, with the cessation of smallpox vaccination programs, the relative partial immunity against MPV has waned and this may have contributed to its re-emergence in endemic African countries [5].

MPOX, originally a zoonotic disease, is transmitted via direct contact with infected animals’ blood, bodily fluids, cutaneous/mucosal lesions, or indirect transmission from eating inadequately cooked meat or other animal products. Human-to-human transmission has been described for MPOX but in a very limited way, and is usually seen in endemic areas of Africa [6]. Close contact with human respiratory secretions (usually prolonged face-to-face contact), skin lesions, or recently contaminated objects will put healthcare workers (HCWs), household members, and other close contacts of the infected individuals at risk [7]. Although the prospect of sexual transmission was not definitively established, four monkeypox-positive cases in Italy raised that possibility since the viral DNA was found in their seminal fluid samples [8]. Another study supported virus shedding in bodily fluids such as saliva, semen, urine, and feces [9].

With the recent emergence of cases in non-endemic countries, the initial WHO assessment did not consider MPOX a public health emergency of international concern (PHEIC) [10]. However, in a subsequent assessment, the WHO then declared MPOX to be a PHEIC [11,12]. In newly affected countries, this is the first time that most of the cases have been confirmed among men who have had recent sexual contact with new or multiple male partners, and clinical presentation has been reported with atypical symptoms, including anogenital and mucosal lesions [6]. MPOX has been historically described mainly in Sub-Saharan African countries, but, more recently, it has been reported in multiple countries in the form of outbreaks. Assessment of the standard of knowledge is critical to ascertain the responses of the general public and HCWs to alerts, especially regarding conditions of an infectious nature, in order to deliver high-quality care by HCWs and ensure compliance by both HCWs and the general public with healthy practices to prevent disease acquisition and spread.

This study aimed to assess the knowledge of the Saudi Arabian public and HCWs about MPOX and their information-seeking attitude during the first month of the WHO alert regarding MPOX, before any cases were yet reported in the Kingdom of Saudi Arabia (KSA), as the first MPOX was reported by the Ministry of Health (MOH) on 14 July 2022 [13].

## 2. Methods

An online survey of HCWs and the public in KSA was conducted from 27 May to 5 June 2022. Participants were invited by convenience sampling techniques via various social media platforms (Twitter and WhatsApp groups) and email lists. Participants were invited to complete the online survey through the SurveyMonkey© (Momentive Inc., San Mateo, CA, USA) online platform, with each response allowed once from each unique IP address to ensure single entries. The first page of the survey included the IRB approval, requested consent regarding participation, explained the study research objectives, and assured confidentiality.

The survey tool was adopted from our previously published research on COVID-19, with modifications related to the new MPOX outbreak [14,15,16,17,18,19,20]. The final version was tested for content validity by our assigned research experts and was piloted with ten HCWs for clarity and consistency. Modifications were implemented, based on the experts’ recommendations. The research team approved the final version of the survey for language accuracy, clarity, and content validity.

The variables surveyed included participants’ sociodemographic and job-related characteristics, COVID-19 infection status, and advocacy for MPOX vaccination, which were reported along with their outcomes in separate publications for both the public and HCWs [18,19]. Multiple questions assessing the participants’ knowledge related to MPOX and MPV in terms of transmission, vaccination information by HCWs, and required isolation precautions. Finally, Generalized Anxiety Disorder (GAD7) [21,22], a self-reported, 7-item validated scale, was used to measure anxiety. We then assessed the independent variables associated with participants’ attitudes to seek more information about MPOX and the variables associated with the knowledge score. (The study questionnaire is attached in the supplementary file).

### 2.1. Sample Size

Using the sample size calculator for proportion, and assuming that 50% of the sample will demonstrate sufficient monkeypox knowledge, the minimum desired sample size for each group that would be required to detect a true proportion of participants with sufficient knowledge, with a confidence level of 95% and a margin of error of 5%, was estimated to be equal to 384 subjects in each group. However, the achieved sample size comprised 1130 HCWs and 1546 public participants. Each sample was statistically analyzed separately.

### 2.2. Statistical Data Analysis

Means and standard deviations were used to describe the continuous or ordinal measured variables, while frequencies and percentages were used for categorically or ratio-measured variables. The histogram and the Kolmogorov–Smirnov tests were applied to test the assumption of normality, and Levene’s test was used to test the homogeneity of variance statistical assumption. Cronbach’s alpha test was used to assess the internal consistency of the measured questionnaires. Multivariable binary logistic regression analysis was used to assess the statistically significant predictor independent variables for specific binary measured outcomes. The association between predictor independent variables and the binary categorically measured variables was assessed with multivariate logistic regression analysis, which was expressed via an adjusted odds ratio (OR) with the associated 95% confidence intervals. While multivariable linear regression analysis was applied to the continuous measured dependent outcome variables and the association between the predictor variables with these continuous measured variables was expressed as beta coefficients, with their associated 95% confidence intervals. The SPSS IBM statistical computing software, version number 21, was used for the statistical data analysis. The statistical alpha significance level was considered to be at the 0.050 level.

Ethical approval was granted by the institutional review board (IRB) at King Saud University (22/0416/IRB).

## 3. Results

A total of 1546 participants completed the public survey. Their baseline characteristics are reported in detail in our previous publication, which addressed their monkeypox worries and vaccination attitude [19]. Table A1 in the Appendix A.2 displays details of MPOX knowledge assessment, which was assessed using seven questions. Overall, 48.7% of respondents knew that the disease could be transmitted before skin blisters appear, and 46.5% knew that it could be transmitted via sexual contact. Regarding MPOX treatment, 47.3% knew that there are effective antiviral medications, while 48% falsely answered that there are none. In addition, 56.6% falsely believed that the old smallpox vaccine is not effective against MPOX, while 36.4% knew that it is. Due to the convergence of names and possible confusion, we assessed the participants’ perceptions as to whether the chickenpox vaccine might be effective against MPOX; 14.7% did not think it is, 64.7% were unsure, and 20.6% falsely thought that it is.

**Figure A1:**
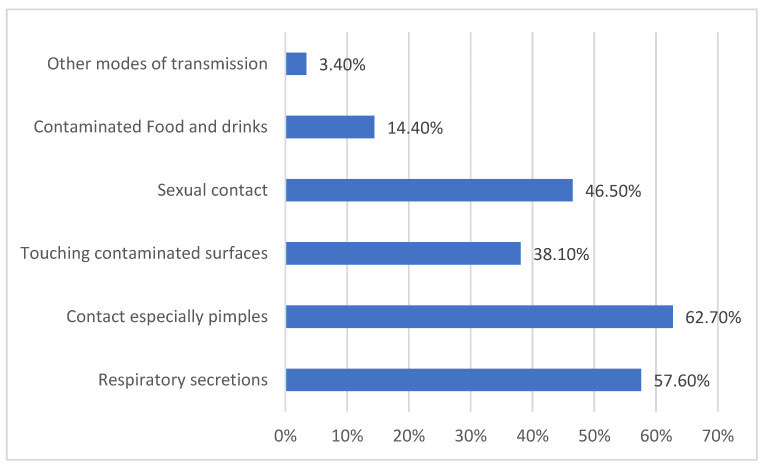
Public perceptions of monkeypox disease transmission modes. (KSA, 27 May to 5 June 2022.)

Regarding MPOX transmission modes, 62.7% of the public correctly reported that the pimples are infective, 57.6% correctly reported that respiratory secretions are also infective, while only 46.5% correctly reported that sexual contact and intimate relationships can be modes for transmission. However, only 38.1% had correctly reported that touching surfaces contaminated with MPV is infective, and 14.4% incorrectly reported that contaminated foods and drinks are possible modes of transmission. Overall, 3.4% of respondents believed that other modes of transmission might exist (Appendix A, Figure A1).

The public participants’ overall mean knowledge score of MPOX was 4.88/9 points, SD = 1.50 points, highlighting a moderate level of knowledge. The knowledge score was dichotomized into low versus high, based on the average score; the results showed that 56% demonstrated relatively high (above-average) MPOX knowledge. 

When considering the public participants’ sources of information about MPOX, 45.6% relied on local official sources, including the MOH, while 28% used international official health websites, such as the Centers for Disease Control and Prevention (CDC) and WHO websites, but 66.1% relied on social networking and other internet-based sources. In total, 61.3% of the participants perceived a need to seek more information about MPOX.

To assess the variables associated with the public participants’ high MPOX knowledge scores, we ran a Multivariate Logistic Binary Regression Analysis. As shown in Table 1, age, employment status, and monthly household income did not correlate significantly with the odds of a high knowledge score. However, respondents holding a university degree or other high educational levels were found to have significantly high scores (OR 1.35; *p*-value = 0.002). Respondents who had previously developed COVID-19 disease had slightly but not significantly lower odds of a high score (OR 0.816; *p*-value = 0.060). Conversely, the respondents who had worries about themselves or their families contracting MPOX had significantly higher knowledge scores (OR 1.843; *p* < 0.001). All the respondents’ sources of information correlated significantly with the odds of high knowledge scores, but with variable significance: local official sources, such as the MOH (OR 2.274; *p* < 0.001), social media channels (OR 1.735; *p* < 0.001), and official international sources, such as the CDC and WHO (OR 1.583; *p*-value = 0.001).

We assessed the public participants’ perceived need to seek more information about MPOX after completing the current survey; 61.3% did show interest in seeking more information. Table 2 highlights the public participants’ characteristics associated with the perceived need to seek more information about MPOX. Males were significantly less likely to seek more information (42.2% less likely; *p*-value = 0.003). Age and educational level did not correlate significantly with their interest in seeking more information, while the household’s income (HHI) >= 15,000 SR per month was associated significantly with less interest (9.6% less interest; *p*-value = 0.010). Participants who had previously developed COVID-19 were found to be significantly less inclined to seek more information about MPOX (39.8% less inclined; *p*-value = 0.002).

However, those who perceived MPOX as virulent and dangerous were significantly more inclined to seek more information (55.9% more inclined; *p* < 0.001), in addition to those who were worried about a possible national lockdown related to MPOX (43.8% more worried; *p*-value = 0.008), echoing those who had moderate to high compliance with universal precautionary measures (9.3% more compliant; *p*-value = 0.044). The participants’ MPOX average knowledge score and GAD7 scores did not correlate significantly with their perceived need to seek more information, while those who supported vaccination against the disease were significantly more inclined to seek more information (67.4% more inclined; *p* < 0.001). The use of social networks and other unofficial sources of information were significant predictors of being less inclined to seek more information about MPOX (58.2% less inclined; *p*-value = 0.001).

In total, 1130 HCWs participated in the survey. Their baseline characteristics are reported in detail in our previous publication, which discussed their monkeypox worries and vaccination attitude [18]. We assessed their MPOX knowledge using four domains: vaccination, transmission modes, clinical presentation, and isolation precautions. Table A2 (in the Appendix A.2) details the descriptive analysis of their responses. Of these, 61.9% of the participants correctly answered that MPOX is caused by a Pox-family virus. Regarding vaccination, only 28.3% correctly answered that the Jynneos vaccine has dual activity against both smallpox and MPOX. Interestingly, 79.7% incorrectly answered that the VARIVAX vaccine, a chickenpox vaccine, is effective against MPOX, while only 34.7% correctly answered that HCWs could benefit from post-exposure prophylaxis in terms of MPOX cases with the smallpox vaccine.

Regarding the HCWs’ knowledge about MPOX’s modes of transmission, nearly half correctly answered that animal-to-human transmission exists; most of them (64.8%) correctly agreed that human-to-human transmission via direct skin contact is a possible mode of transmission, while 67.6% correctly answered that MPOX is not an airborne transmissible disease. In comparison, only 53.7% correctly agreed that it is transmitted via droplets. In total, 94.4% correctly reported that it is not a foodborne disease, and 96% believed falsely that other modes of transmission might indeed exist. Regarding the clinical presentation of MPOX, only 57.5% correctly answered that it has similar symptoms to COVID-19 before the appearance of the rash. The majority correctly indicated that fever, rash, and headaches are indeed the presenting symptoms of MPOX. At the same time, less than half of the HCWs correctly reported that myalgia and lymphadenopathy are MPOX symptoms. However, the majority (>90%) of the participants had the misconception that respiratory distress, hemodynamic shock, seizures, and loss of the sense of smell, as well as acute kidney injury, are among the clinical manifestations of MPOX. Regarding isolation precautions against MPOX, 73.6% correctly indicated that contact precautions are needed, while 65.2% incorrectly answered that airborne precautions might be needed, in comparison to 53.8% who correctly answered that droplet precautions are needed at a certain phase of the disease; 96% incorrectly reported that other isolation precautions are required to control the disease transmission.

Table A3 in the Appendix A.2 displays the HCWs’ dissected knowledge score. The highest achieved score was regarding MPOX precautionary modes score (mean score 2.9/4, 72.5%, SD 0.8), followed by transmission modes (mean score was 6.2/9, 68.9%, SD 1.5). The overall HCWs’ MPOX knowledge showed a mean score of 14.4/28, 51.6%, and SD of 3.8.

**Figure A2:**
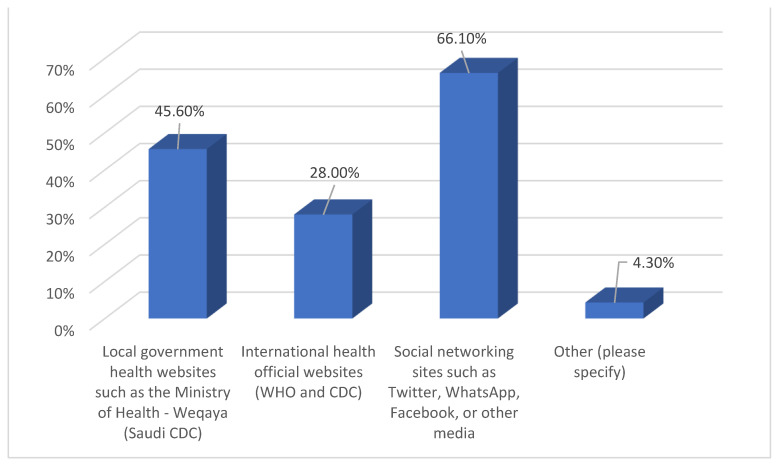
Public sources of information about monkeypox disease. (KSA, 27 May to 5 June 2022.)

**Figure A3:**
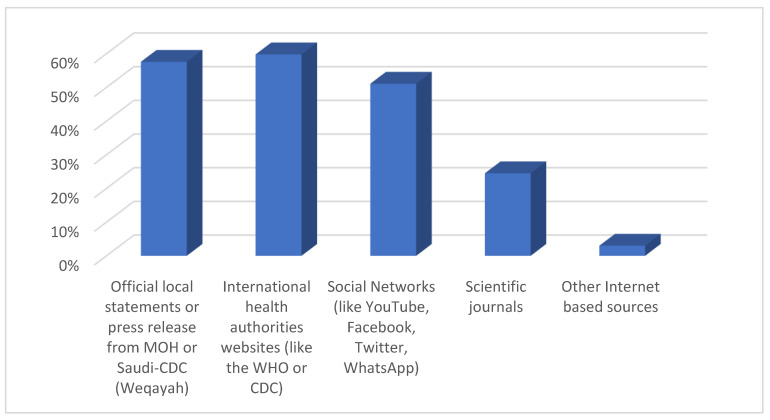
HCWs’ sources of information about monkeypox disease. (KSA, 27 May to 5 June 2022.)

Sources of information on MPOX for the public and HCWs are displayed in Figure A2 and Figure A3 of the Appendix A. Most HCWs (74.2%) perceived the need to seek more information about MPOX after receiving the survey. Overall, 57.6% used official local sources, 59.8% used official international health authorities’ sources, and 51.1% used social media and internet-based networks, while 24.5% only relied on scientific journals. 

Table 3 shows the variables associated with the HCWs’ perceived need to seek more information about MPOX after involvement in the current survey. HCWs’ sex, age, working hospital type, self-rated awareness level of MPOX, participants’ GAD7, and overall MPOX knowledge score did not correlate significantly with their odds of seeking more information about MPOX.

Expatriate HCWs had significant odds of seeking more information about MPOX (4.10 times more likely, *p* < 0.001). HCWs’ worry about MPOX progress to a pandemic was a significant positive predictor of their perceived need to read more about it (OR = 1.243, *p*-value = 0.018). Their worry about international travel restrictions due to MPOX was also a significant positive predictor (OR = 1.57; *p* < 0.001). Echoing that finding, their perception of MPOX as a severe and virulent disease was another strong positive predictor (OR = 1.359; *p*-value = 0.009). The strongest predictor to seek more information about MPOX was their perception of the need to apply tighter infection control prevention measures to control the progress of the disease (1.901 times more, *p* < 0.001). HCWs with a household size of >= 4 persons had a high perceived need to seek more information regarding MPOX (OR = 1.81, *p* < 0.001), in addition to those who had a monthly income >= 15,000 (OR = 1.282, *p* < 0.010).

To understand the correlation of HCWs’ characteristics with their assessed knowledge score, we ran a multivariate linear regression analysis, as shown in Table 4. Male HCWs had significantly lower mean knowledge scores compared to females (β coefficient = −0.564; *p*-value = 0.007), while age did not correlate significantly with their knowledge score *p* = 0.146. Physicians, compared to other HCWs, were found to be significantly more informed about the disease (β coefficient = 875; *p* < 0.001). HCWs’ self-rated high awareness of MPOX correlated positively and significantly with their knowledge score about MPOX (β coefficient = 0.741; *p* < 0.001). Use of any source of information about MPOX correlated significantly with a higher knowledge score compared to using no source at all (β coefficient = 7.323; *p* < 0.001). HCWs’ household monthly income correlated positively and significantly with their MPOX knowledge score (β coefficient = 0.133; *p*-value = 0.045). Interestingly the HCWs’ perception of the need to seek more information about MPOX did not correlate significantly with their knowledge score (*p*-value = 0.098). In terms of the participants’ working institution type, those who worked in secondary and tertiary centers had significantly higher mean MPOX knowledge scores (β coefficient = 0.700 and 0.918; *p*-values 0.021 and <0.001, respectively), while participants working in the operating rooms, outpatient department, and general hospital wards had significantly lower mean MPOX knowledge scores compared to those working in other units (β coefficient = −1.135, −0.696, and −0.564; *p*-values of 0.047, 0.002, and 0.016, respectively).

Table 5 shows the correlation between the HCWs’ overall MPOX knowledge score and their other cognitive MPOX-related variables. The overall knowledge score correlated significantly and positively with their self-rated awareness of MPOX and their self-rated worry about MPOX progress to a pandemic (rho = 0.253 and 0.260; *p* < 0.010 and *p* < 0.050, respectively). On the other side, the HCWs’ GAD7 score correlated weakly and negatively with their MPOX knowledge score, but positively and significantly with their worry about MPOX progressing to a pandemic and their perception of it as a severe and virulent disease (rho = 0.279 and 0.200; *p* < 0.010, respectively). The HCWs’ worry about MPOX progressing to pandemic status correlated significantly and positively with their perception of MPOX as a virulent and severe disease (rho = 0.319; *p* < 0.010).

## 4. Discussion

Knowledge dictates attitudes and practices, but compliance with knowledge is needed to assure the achievement of intended goals; in our case, dealing with infectious disease and preventive measures [23]. Additionally, the sources of that knowledge will affect its accountability, trustworthiness, and, therefore, its application [24]. Preparedness regarding COVID-19 has affected its outcomes differently across the world; therefore, facing future pandemics needs the planning of resources and the learning of lessons from the previous pandemic from COVID-19. MPOX has been considered a zoonotic disease, but its emergence in humans a few decades ago alerted healthcare systems to the fact that zoonotic disease might become an alarming threat that will endanger humanity in a similar way to the Ebola and avian flu viruses [25,26]. The MPOX literature still has gaps that remain critical to its understanding and the better education of healthcare stakeholders [22].

The recent sudden global rise in human-to-human transmission of MPOX warrants careful attention. Despite detecting only three positive cases in the Saudi community, all coming from Europe [13], our sampled population from the HCWs and the general public showed a moderate level of awareness about the disease, even before local cases were reported.

The HCWs in our study used international official health sources as their source of MPOX knowledge in 60% of cases, while the public used it in 28% of cases, which can be explained by the HCWs’ educational and medical qualifications since they also used scientific journals in 24.5% of cases. Both groups used similar local official health sources. The public used social networks more widely than did HCWs (66% compared to 51%), which also stems from the HCWs’ critical appraisal methodology of acquiring knowledge, which discourages them from using sources with low accountability. A previous study on MPOX in Africa reported that media campaigns to raise awareness did not demonstrate effectiveness in changing public health behaviors regarding the disease [27]. Interestingly, in our study, both groups had high rates of the perceived need to seek more information about MPOX, at 61% for the public and 74% for HCWs, which indicates their high alert level and concerns about this re-emerging disease.

We found major gaps in the HCWs’ knowledge about MPOX, compared to previous studies that assessed HCWs’ knowledge. Some gaps are critical, especially regarding vaccination, as the majority did not realize that the Jynneos vaccine has dual activity against smallpox and MPV. The vast majority falsely thought that the VARIVAX vaccine is also effective against MPV [28,29,30]. Such a vaccination knowledge gap might be a challenge for future vaccination campaigns if needed, or it may fuel vaccination hesitancy and limit our ability to contain a pandemic.

Physicians and HCWs working in secondary and tertiary centers had good odds of high MPOX knowledge. Understandably, variations in knowledge would occur among HCWs; those working in secondary or tertiary centers might potentially have a richer knowledge base; this might be explained by their clinical exposure, in addition to their higher expertise and qualifications. MPOX usually presents as a simple viral prodromal disease in primary healthcare facilities; therefore, a deficiency in the knowledge of primary healthcare providers might endanger the community if cases fail to be recognized early. In a previous study in 2020, general practitioners who worked in community health centers had higher levels of knowledge than those who worked in private clinics [28]. Another study showed that among HCWs in Italy, there were substantial knowledge gaps in relation to MPOX [29]. Female HCWs and those who rated themselves as having high self-awareness about MPOX had good odds of a high knowledge score, which translates to their personality type presenting augmented anxiety regarding illnesses in general, compared to males, and, therefore, tend to enrich their medical knowledge, especially that of a preventive nature.

Among the general population, age, employment status, and monthly household income did not correlate with MPOX knowledge scores. In contrast to this finding, a study from Saudi Arabia showed that these factors were correlated positively with the level of knowledge of MPOX, in addition to the participants’ marital status [30].

In relation to the public responses, the variables associated with the odds of a high knowledge score partly echoed the HCWs’ variables, as the use of all different sources of information by the public and HCWs was correlated significantly with the odds of high knowledge scores. Interestingly, those who perceived the need to seek more information about MPOX did not have significant odds for either low or high knowledge scores, which reflects their perceived low knowledge base and their need to enrich it. As expected, members of the public holding university degrees and higher educational levels scored high knowledge scores, which echoes our colleagues’ results [30]. Additionally, public participants who had personal and family worries about contracting the disease had significantly high knowledge scores, which is again an expected intuitive healthy behavior since worry dictates the perception of risk, which translates into preventive practice, stemming from reading and acquiring targeted knowledge [31].

Both members of the public and HCWs who showed an interest in reading more about MPOX overlapped by either worry about MPOX progressing into a pandemic or national lockdown, or it causing international travel restrictions; our colleagues Meo et al. also detected a similarly high level of public alert and worry regarding MPOX progress and its spread worldwide [32]. HCWs who perceived MPOX as being potentially severe and virulent, and those who perceived the need to apply tighter infection control prevention measures, also perceived a need to seek more information about MPOX. The general public’s responses almost mirrored the HCWs’, as public participants who perceived MPOX as being virulent and dangerous, along with those who had moderate to high compliance with universal precautionary measures, also showed high interest in reading more about MPOX. Male participants from the public group were less inclined to seek more information about MPOX. Interestingly, those who had already developed COVID-19 also showed less interest in seeking more information, which can be explained by their feeling of having passed through a universal pandemic safely, which can give reassurance throughout the current alert as they probably have more optimism regarding the future [33].

Conversely, this was not the case for those public participants who supported vaccination against MPOX, and who showed significantly high interest in reading more about the disease, reflecting their high alert level. Additionally, the public’s use of non-official sources of information about MPOX was significantly associated with less inclination to increase their MPOX knowledge base; this might be explained by the impressively widespread application and usage of those sources as a repository of information for patients and their families through support and e-learning [34,35].

Our public participants understood MPOX modes of transmission fairly well, even regarding sexually intimate relationships. Our results were largely consistent with those reported from another local questionnaire issued by Alshahrani et al. in a study with 480 participants from the general population [30].

The knowledge gap and the incorrect answers could be attributed to the scarcity of cases within the community, and the lack of interest in pursuing more information regarding MPOX from some members of the public. Another possible factor could be misleading and untrustworthy social media reports and other internet resources, as most of our participants (66.1%) considered them their primary reference. COVID-19 fatigue still overshadows both international and national scenes, which could potentially make people less interested in acquiring information about the current MPOX outbreak. Nonetheless, nearly two-thirds of the public participants (61.3%) felt the need to seek out more information regarding MPOX.

The HCWs’ and the public’s knowledge regarding the transmission modes were not vastly different. Only half of the HCWs knew that droplet precautions might be needed at a certain phase of the disease. Similarly, an Italian study addressing HCWs’ knowledge level showed that most participants recognized the potential transmission of MPOX through the respiratory system via respiratory droplets, direct contact, and body fluids, and that standard preventive measures may be sufficient to avoid infection (74.8%) [29].

Although the HCWs are at high risk of contracting the disease from symptomatic undiagnosed patients, a couple of hurdles could be identified as the causes of their struggle to gain sufficient information regarding MPOX. One influential factor is the limited awareness campaigns that were organized by local and international health authorities, such as the WHO, aiming to familiarize the HCWs with the re-emerging disease, although limited research showed that previous MPOX campaigns were less effective in raising awareness [27]. Other factors could be insufficient access to free, trustworthy scientific resources and, finally, a lack of time. Additionally, with many healthcare authorities still employing strict isolation precautions and infection control measures because of the COVID-19 pandemic, many HCWs might feel that they are already taking sufficient precautions against other infectious diseases.

In comparison with previously published research [29,30], our study is the first to explore and compare HCW and public MPOX knowledge in one study in Saudi society, early in the response to the WHO MPOX alert. Both groups showed a decent attitude toward seeking more MPOX knowledge, which correlated positively with their worry about and awareness of the disease. These observations are mostly as a consequence of the remitting COVID-19 pandemic, which encouraged members of the public and HCWs to garner more information about any emerging disease. The knowledge gaps among HCWs were evident in the clinical presentation and vaccinations, which gaps need addressing in order to contain emerging MPOX cases. Exploring the public’s and HCWs’ MPOX vaccination advocacy is an important step in limiting the disease’s spread if it becomes a worldwide or local pandemic or if it spreads in certain communities. The COVID-19 pandemic has hit the human race hard over the last 2 years; its power partly stemmed from it being a novel disease, coinciding with limited public and HCW knowledge of its characteristics, which limits society’s potential to contain and fight the disease transmission and the fallacies related to vaccination. Therefore, the employment of strategies learned during the COVID-19 pandemic should be strategically integrated into the healthcare system in order to limit and contain any future emerging infectious diseases [25,26,36,37,38,39,40].

The limitations of our results are related to our limited ability to analyze both groups into subgroups, based on their recent traveling experience and social activities. Other limitations stem from the study’s observational nature and the limitations of similar studies, in relation to their sampling technique and this being a knowledge assessment study; the responses are, therefore, liable to recall bias. As no other sampling method was feasible during the rapidly evolving monkeypox surge, convenience sampling was used to develop our hypotheses and objectives for use in the future, to ensure more rigorous research studies [41].

## 5. Conclusions

We identified areas of improvement in both HCWs’ and the public’s MPOX awareness and knowledge gaps, especially the HCWs. Both groups showed a decent attitude to seeking out more MPOX knowledge, which correlated positively with their worry about and awareness of the disease. Policymakers should employ such an attitude in awareness campaigns to prevent the spread of the disease and encourage vaccination in case it is needed. The limitations of our study, namely, its observational nature and possible recall bias, demonstrate the need for further future research into such emerging infectious disease threats.

## Figures and Tables

**Table 1:** Multivariate binary logistic regression analysis of the general public’s odds of high MPOX knowledge score. (KSA, 27 May to 5 June 2022.)

Variable	Multivariate Adjusted Odds Ratio (OR)	95% CI for OR		*p*-Value
		Lower	Upper	
Age group	0.975	0.874	1.087	0.646
Educational Level University degree or higher	1.358	1.118	1.649	0.002
Employment status Employed	1.172	0.935	1.467	0.168
Previously developed COVID-19	0.816	0.660	1.008	0.060
Monkeypox infection personal and family worry	1.843	1.486	2.286	<0.001
Monkeypox information source				
MOH	2.274	1.772	2.919	<0.001
CDC and WHO websites	1.583	1.215	2.064	0.001
Social media	1.735	1.332	2.262	<0.001
Other internet sources	1.757	1.001	3.082	0.049
Monthly Household Income	1.064	0.990	1.143	0.094

DV = High MPOX knowledge score; OR = odds ratio; CI = confidence interval; MOH = Ministry of Health; CDC = Centers for Disease Control and Prevention; WHO = World Health Organization.

**Table 2:** Multivariate binary logistic regression analysis of the public’s variables associated with high odds of seeking more information about monkeypox. (KSA, 27 May to 5 June 2022.)

Variable	Multivariate Adjusted Odds Ratio (OR)	95% CI		*p*-Value
		Lower	Upper	
Sex Male	0.578	0.402	0.832	0.003
Age group	0.965	0.855	1.088	0.557
Educational Level	0.933	0.756	1.151	0.518
Households’ Monthly income 15,000 SR/Month	0.904	0.838	0.976	0.010
Previously developed COVID-19	0.602	0.435	0.832	0.002
Perceives MPOX as dangerous and virulent	1.559	1.243	1.954	<0.001
Family compliance with universal precautionary measures Moderate or higher compliance	1.093	1.002	1.192	0.044
Monkeypox knowledge score	1.029	0.954	1.110	0.463
GAD7 score	0.997	0.976	1.018	0.772
Supports vaccination against MPOX	1.674	1.338	2.095	<0.001
Reliance on social networks and nonofficial sources of information	0.418	0.247	0.709	0.001
Worried about a possible national lockdown due to MDP	1.438	1.099	1.881	0.008

DV = perception of the need to seek more information about MPOX; OR = odds ratio; CI = confidence interval; MPOX = monkeypox disease; GAD = generalized anxiety disorder.

**Table 3:** Multivariate binary logistic regression analysis of the HCW’s MPOX information-seeking behavior. (KSA, 27 May to 5 June 2022.)

	Multivariate Adjusted Odds Ratio (OR)	95% C.I.		*p*-Value
		Lower	Upper	
**Sex** **Male**	0.959	0.669	1.373	0.819
**Age**	1.000	0.983	1.018	0.964
**Nationality** **Non-Saudi**	4.064	2.726	6.057	<0.001
**Hospital Type** **Tertiary**	0.821	0.666	1.012	0.064
**Self-rated awareness of MPOX**	1.040	0.876	1.235	0.652
**Worry that MPOX will progress to a pandemic**	1.243	1.038	1.488	0.018
**MPOXMPOX perception as severe and virulent**	1.359	1.079	1.712	0.009
**(GAD7) score.**	0.992	0.955	1.031	0.683
**Monkeypox overall knowledge score**	1.046	0.993	1.102	0.091
**Agreement with the need for tighter infection control measures**	1.901	1.477	2.447	<0.001
**International travel restrictions due to MPOX worry**	1.570	1.140	2.161	0.006
**Household size (family members)** **>=4 persons**	1.807	1.465	2.229	<0.001
**Households’ monthly income level** **>=15,000 SR**	1.282	1.140	1.442	<0.001

DV = high perceived need to seek more information about monkeypox disease. SR = Saudi Riyal (currency); MPOX = monkeypox disease; OR = odds ratio; CI = confidence interval; GAD7 = generalized anxiety disorder.

**Table 4:** Multivariate linear regression analysis of the HCWs’ variables associated with their mean overall MPOX knowledge score. (KSA, 27 May to 5 June 2022.)

Variable	Unstandardized Beta Coefficients	95.0% CI for Beta Coefficient		*p*-Value
		Lower Bound	Upper Bound	
Sex Male	−0.564	−0.976	−0.153	0.007
Age	0.015	−0.005	0.034	0.146
Clinical Role Physicians	0.875	0.428	1.321	<0.001
MPOX Self-rated awareness	0.741	0.565	0.916	<0.001
Use of any source of information	7.323	6.468	8.177	<0.001
Households Monthly Income	0.133	0.003	0.263	0.045
Perception of the need to seek information about MPOX	0.402	−0.074	0.878	0.098
GAD7 score	−0.043	−0.083	−0.003	0.035
Hospital type Secondary	0.700	0.107	1.293	0.021
Hospital type Tertiary	0.918	0.446	1.390	<0.001
Working unit Operating Room	−1.135	−2.257	−0.013	0.047
Working Unit Outpatient area	−0.696	−1.138	−0.254	0.002
Working unit General hospital wards	−0.564	−1.022	−0.107	0.016

DV = Monkeypox overall knowledge score. Model overall significance: f (13,1113) = 45.54, *p* < 0.001. Model R = 0.589, Adj. R-squared = 0.34. MPOX= monkeypox disease; OR = odds ratio; CI = confidence interval.

**Table 5:** Bivariate correlations between HCWs’ MPOX knowledge, awareness, perception of severity, and worry about its progress to pandemic status. (KSA, 27 May to 5 June 2022.)

Variable	Knowledge Score	MPOX Awareness	Worry of MPOX Progress to Pandemic	Perception of MPOX as Severe and Virulent
MPOX overall knowledge score	1.000			
HCWs’ self-rated awareness of MPOX	0.253 **			
Worry of MPOX progress to pandemic	0.076 *	0.260 **		
Perception of MPOX as severe and virulent	−0.056	−0.040	0.319 **	
GAD7 score	−0.059 *	−0.004	0.279 **	0.200 **

* Correlation is significant at the 0.050 level; ** correlation is significant at the 0.010 level. MPOX = monkeypox disease; GAD7 = generalized anxiety disorder.

## Data Availability

Data is available from the corresponding author upon reasonable request.

## Supplementary Materials

The following are available online at https://www.mdpi.com/article/10.3390/vaccines10122071/s1, File S1: The study questionnaire.

## Abbreviations

CDC	Centers for Disease Control and Prevention
COVID-19	Coronavirus disease 2019
GAD7	Generalized Anxiety Disorder 7-item
HCW	Healthcare workers
MOH	Ministry of Health
MPV	Monkeypox virus
MPOX	Monkeypox disease
PHEIC	Public Health Emergency of International Concern
SARS-CoV-2	Severe acute respiratory syndrome coronavirus 2
WHO	World Health Organization

## Appendix A

### Appendix A.2. CDC Center of Disease Control, MOH Ministry of Health, the WHO World Health Organization

**Table A1:** The public’s monkeypox knowledge assessment questions (KSA, 27 May to 5 June 2022.)

**Can Monkeypox be transmitted before symptoms appear, especially skin blisters?**	**No.**	**Frequency**
No	793	51.3
Yes	753	48.7
**Do you know if there is a cure for monkeypox?**		
Unsure	72	4.7
There is no effective treatment, treatment is poor	742	48
There are antivirals to treat this disease	732	47.3
**Do you think that the Smallpox vaccine used before 1980 is effective against monkeypox?**		
No	108	7
Unsure	875	56.6
Yes	563	36.4
**Do you think that the chickenpox vaccine is effective against monkeypox?**		
No	227	14.7
Unsure	1000	64.7
Yes	319	20.6
**Overall monkeypox disease knowledge score, mean (SD).** **maximum possible score = 9**		4.88 (1.49)
Low	681	44
High	865	56
**Perceived need to seek more information about monkeypox disease**	948	61.3

**Table A2:** HCWs’ MPOX knowledge assessment questions analysis. (KSA, 27 May to 5 June 2022.)

	Best Correct Answer	Incorrectly Answered No. Frequency	Correctly Answered No. Frequency
**Jynneos is an FDA-approved vaccine for the prevention of Monkeypox disease with dual activity against Smallpox and Monkeypox infections.**	True.	808 (71.7)	319 (28.3)
**Chickenpox vaccines, such as Varivax, have dual activity against mhickenpox and monkeypox infections.**	False.	898 (79.7)	229 (20.3)
**HCW exposed to monkeypox case benefit from receiving post-exposure prophylaxis (PEP) with the smallpox vaccine**	True.	736 (65.3)	391 (34.7)
**Monkeypox is caused by the Pox family virus**	True	429 (38.1)	698 (61.9)
**Monkeypox disease transmission**			
Animal to human	True.	565 (50.1)	562 (49.9)
Human to human via direct skin contact	True.	397 (35.2)	730 (64.8)
Human to human via sexual route	True.	668 (59.3)	459 (40.7)
Airborne	False.	366 (32.5)	761 (67.6)
Droplet	True.	522 (46.2)	605 (53.7)
Food-Borne	False.	63 (5.6)	1064 (94.4)
Contaminated water	False.	98 (8.7)	1029 (91.3)
Other modes	False.	45 (4)	1082 (96)
**Monkeypox disease presenting signs and symptoms**			
Before the rash appears, the symptoms of COVID-19 and Monkeypox are very similar.	True.	479 (42.5)	648 (57.5)
Fever	True.	186 (16.5)	941 (83.5)
Rash	True.	173 (15.4)	954 (84.6)
Headaches	True.	424 (37.6)	703 (62.4)
Lymphadenopathy	True.	599 (53.1)	528 (46.9)
Myalgias	True.	612 (54.3)	515 (45.7)
Exhaustion-Physical	True.	804 (71.3)	323 (28.7)
Respiratory distress	False.	265 (23.5)	862 (76.5)
Shock-Hemodynamic instability	False.	57 (5.1)	1070 (94.9)
Seizures	False.	57 (5.1)	1070 (94.9)
Loss of the sense of smell	False.	61 (5.4)	1066 (94.6)
Acute kidney injury	False.	47 (4.2)	1080 (95.8)
**Monkeypox required isolation methods**			
Contact precautions	True.	298 (26.4)	829 (73.6)
Airborne precautions	False.	392 (34.8)	735 (65.2)
Droplet precautions	True.	521 (46.2)	606 (53.8)
Other isolation techniques	False.	24 (2.1)	1103 (97.9)

**Table A3:** HCWs’ knowledge scores. (KSA, 27 May to 5 June 2022.)

Knowledge Score	Mean Score	Mean % Score	SD	IQR	Maximum Score
**MPOX transmission modes’ knowledge score**	6.2	68.9	1.5	2.0	9 points
**MPOX vaccination knowledge score**	1.0	33.3	0.9	2.0	3 points
**MPOX clinical presentation knowledge score**	4.5	35.4	2.2	3.0	12 points
**MPOX precautionary methods knowledge score**	2.9	72.5	0.8	1.0	4 points
**Overall MPOX knowledge score**	14.4	51.6	3.8	5.0	28 points

SD = standard deviation, IQR = interquartile range; MPOX = monkeypox disease.
