# Supplementary material for: Monkeypox Disease (MPOX) Perceptions among Healthcare Workers versus General Population during the First Month of the WHO Alert: Cross-Sectional Survey in Saudi Arabia
Source: Vaccines (Basel). 2022 Dec 3;10(12):2071. doi: 10.3390/vaccines10122071 (PMC9785604; doi:10.3390/vaccines10122071)
Supplement: Supplementary file 1 [file vaccines-10-02071-s001.zip › File S1-Monkeypox HCWs w keys.pdf]

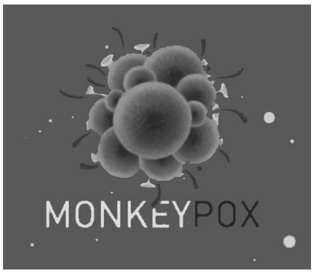

Monkeypox versus COVID-19 perceptions among HCWs in Saudi Arabia

* 1. **Greetings dear healthcare provider,**

With the Monkeypox coming soon after the COVID-19 pandemic, we would like to explore our healthcare workers (HCWs) valuable input on this topic: to compare the recent Monkeypox outbreaks in several countries with the COVID-19.

Kindly take 5 minutes in the following survey, keeping in mind that all your answers are confidential.

The study was approved by the Institutional Review Board at the College of Medicine, King Saud University (approval # 22/0416/IRB).

You can choose to provide (or not to provide) your email at the end of the survey, and it will be strictly confidential and used to choose the Lucky Draw Winners for Amazon coupons.

Thank You!

The Emerging Infectious Disease Research Consortium

med.researcher.2020@gmail.com

- ◯ I am a healthcare worker in Saudi Arabia, and I **ACCEPT** to participate in this Survey
- ◯ I do **NOT accept** to participate in this Survey

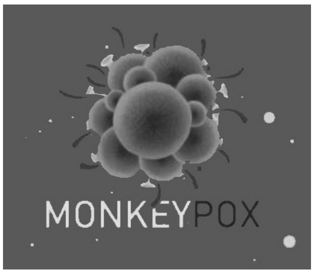

Monkeypox versus COVID-19 perceptions among HCWs in Saudi Arabia

Demographics

Please Choose Your Answers then Press "Next"

* 2. You are

- ◯ Consultant
- ◯ Assistant consultant / Fellow
- ◯ Resident / Registrar
- ◯ Nurse
- ◯ Intern
- ◯ Medical student
- ◯ Lab / Radiology Technician
- ◯ Other (please specify)

* 3. What is your age?

Age in years:

* 4. What is your gender?

- ◯ Female
- ◯ Male

* 5. Which of the following best describes your current marital status?

- ◯ Married
- ◯ Single
- ◯ Divorced
- ◯ Widowed

* 6. Nationality:

- ◯ Saudi
- ◯ Expatriate

* 7. At what Hospital area do you work usually most of the time?

- ◯ ICU
- ◯ ER
- ◯ OR
- ◯ Infectious Disease / Isolation wards
- ◯ General wards
- ◯ OPD
- ◯ non-clinical area
- ◯ Other (please specify)

* 8. Your hospital type of practice:

- ◯ Primary healthcare center
- ◯ Secondary-care hospital
- ◯ Tertiary hospital

* 9. Were you previously diagnosed with COVID-19 yourself?

- ◯ No
- ◯ Yes (please specify how many times)

* 10. Have you travelled abroad in the last month to a country where Monkeypox was recently reported?

- ☐ West or Central Africa
- ☐ UK
- ☐ Europe
- ☐ USA
- ☐ Other (please specify where)
- ☐ Canada
- ☐ UAE
- ☐ Australia
- ☐ None of the above

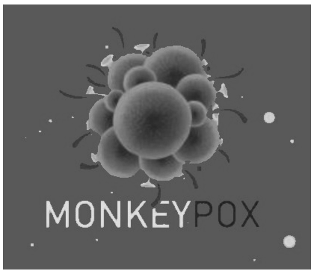

Monkeypox versus COVID-19 perceptions among HCWs in Saudi Arabia

Monkeypox versus COVID-19

* 11. How aware are you of the recent increase in Monkeypox cases worldwide?

- ◯ A great deal
- ◯ A lot
- ◯ A moderate amount
- ◯ A little
- ◯ None at all

* 12. How worried are you that Monkeypox can cause worldwide pandemic similar to COVID-19?

- ◯ A great deal of worry
- ◯ A lot
- ◯ A moderate amount
- ◯ A little
- ◯ None worried at all

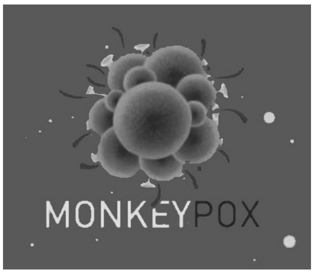

Monkeypox versus COVID-19 perceptions among HCWs in Saudi Arabia

Knowledge about Monkeypox

* 13. As compared to Smallpox: the Monkeypox is causing more severe disease.

- ◯ Agree
- ◯ Neither agree nor disagree
- ◯ Disagree

* 14. Jynneos is FDA-approved vaccine for the prevention of Monkeypox disease with dual activity against Smallpox and Monkeypox infections.

- ◯ True
- ◯ False
- ◯ I do not know

* 15. Chickenpox vaccine, like Varivax, has dual activity against Chickenpox and Monkeypox infections.

- ◯ True
- ◯ False
- ◯ I do not know

* 16. HCW exposed to a case of Monkeypox should receive Post-exposure prophylaxis (PEP) with smallpox vaccine:

- ◯ Agree
- ◯ Neither agree nor disagree
- ◯ Disagree

17. What are the modes of Monkeypox transmission?
(Please select all that apply)

- [ ] animal to human
- [ ] human to human via skin direct contact
- [ ] human to human via sexual route
- [ ] airborne
- [ ] droplet
- [ ] food borne
- [ ] contaminated water
- [ ] Other (please specify)

18. In your opinion:
In case Monkeypox vaccine becomes available, who should be prioritised to receive it ?
(Please choose all that may apply)

- [ ] Healthcare professionals
- [ ] Teachers
- [ ] Elderly aged 65 and over
- [ ] Patients with immune deficiency
- [ ] Patients with chronic disease (like DM, hypertension, renal disease...)
- [ ] College students
- [ ] Inmates and staff of correctional institutions
- [ ] Non-pregnant women of child-bearing age
- [ ] Adolescents and adults living with children
- [ ] International travelers
- [ ] Children
- [ ] Other (please specify)

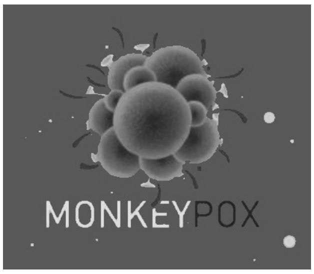

Monkeypox versus COVID-19 perceptions among HCWs in Saudi Arabia

HCW's attitude and perceptions in relation to the new COVID Mutations

* 19. Signs and symptoms of Monkeypox include:
(Choose all that apply)

- [ ] Fever
- [ ] Rash
- [ ] Headache
- [ ] Lymphadenopathy
- [ ] Myalgia
- [ ] Exhaustion
- [ ] Respiratory distress
- [ ] Shock
- [ ] Seizures
- [ ] Loss of smell
- [ ] Acute kidney injury

* 20. What are the isolation precautions for patients suspected to have Monkeypox?

- [ ] Contact precautions
- [ ] Airborne precautions
- [ ] Droplet precautions
- [ ] Other (please specify)

* 21. Before the rash appearance, the symptoms of COVID-19 and Monkeypox are very similar.

- ( ) True
- ( ) False

* 22. Monkeypox is caused by:

- ( ) A virus from Pox family
- ( ) A Chickenpox virus
- ( ) A Smallpox virus
- ( ) It’s not caused by a virus

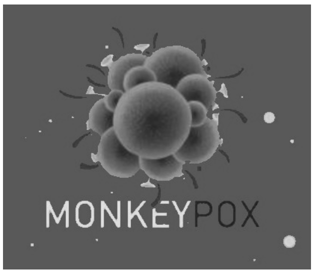

Monkeypox versus COVID-19 perceptions among HCWs in Saudi Arabia

Sources of information about the pandemic

Please Choose Your Answers then Press "Next"

* 23. What is/are your source(s) of information about infectious disease outbreaks (like COVID-19 or Monkeypox)?
(Check all that apply)

- [ ] Official local statements or press release from MOH or Saudi-CDC (Weqayah)
- [ ] International health authorities websites (like the WHO or CDC)
- [ ] Social Networks (like YouTube, Facebook, Twitter, WhatsApp)
- [ ] Scientific journals
- [ ] Others (Please specify)

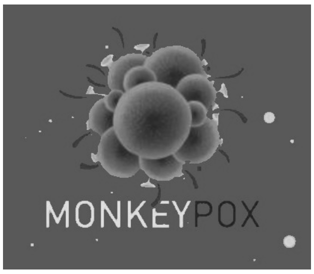

Monkeypox versus COVID-19 perceptions among HCWs in Saudi Arabia

Worries from Monkeypox

* 24. Comparing your worries about COVID-19 versus Monkeypox, which one is more worrisome to you **now**?

- I am worried more about COVID-19
- I am worried more about Monkeypox

* 25. With the new Monkeypox outbreaks in some countries: healthcare workers should apply more infection control measures than the current ones.

Agree	Neither agree or disagree	Disagree
◯	◯	◯

* 26. With the new Monkeypox outbreaks in many countries:
Please rate how much worry about traveling abroad?

Not worried at all	Somewhat worried	Extremely worried
◯	◯	◯

* 27. Over the last 2 weeks, how often have you been bothered by the following problems?

	Not at all	Several days	More than half the days	Nearly every day
Feeling nervous, anxious or on edge	◯	◯	◯	◯
Not being able to stop or control worrying	◯	◯	◯	◯
Worrying too much about different things	◯	◯	◯	◯
Trouble relaxing	◯	◯	◯	◯
Being so restless that it is hard to sit still	◯	◯	◯	◯
Becoming easily annoyed or irritable	◯	◯	◯	◯
Feeling afraid as if something awful might happen	◯	◯	◯	◯

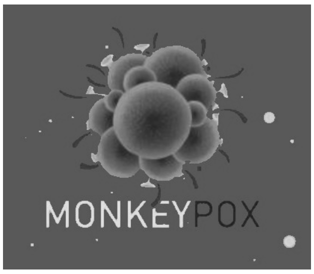

Monkeypox versus COVID-19 perceptions among HCWs in Saudi Arabia

28. Thanks for your valuable input!

Optional: Provide your email address to join our Lucky Draw for Online Coupons:

29. How many persons do you live with?

- ◯ 1-3 members
- ◯ 4-6 persons
- ◯ 7-10 persons
- ◯ >=11

30. Mean family’s monthly income

- ◯ Less than 10000 SR
- ◯ 10001-15000 SR
- ◯ 15001-20000 SR
- ◯ More than 20000 SR
- ◯ Prefer not to answer

31. Did this survey make you more interested to read about Monkeypox?

- ◯ Yes
- ◯ No
