# Supplementary material for: Monkeypox Disease (MPOX) Perceptions among Healthcare Workers versus General Population during the First Month of the WHO Alert: Cross-Sectional Survey in Saudi Arabia
Source: Vaccines (Basel). 2022 Dec 3;10(12):2071. doi: 10.3390/vaccines10122071 (PMC9785604; doi:10.3390/vaccines10122071)
Supplement: Supplementary file 1 [file vaccines-10-02071-s001.zip › File S1-Monkeypox Public Survey w key.pdf]

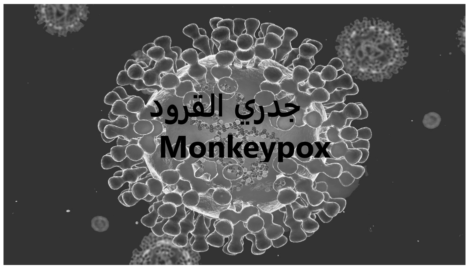

تقييم معلومات البالغين عن جدري القرود مقارنة بالكوفيد

Monkeypox versus COVID-19 Vaccine and Perceptions among Adults and Parents

أنت مدعو للمشاركة في هذه الدراسة عن رأي البالغين بالنسبة لجدري القرود و الكوفيد

الدراسة موافق عليها من لجنة الأبحاث في جامعة الملك سعود و مشاركتك فيها طوعية حيث نهدف من خلالها لمعرفة رأي البالغين او الوالدين بالنسبة لجدري القرود و الكوفيد

اختياري: يمكنك الإشتراك في سحب على كوبونات اونلاين

و لمزيد من المعلومات يمكنك التواصل مع الباحثين الرئيسين
Emerging Infectious Disease Research Consortium
med.researcher.2020@gmail.com

You are invited to participate in this study which assesses the factors affecting adults' perceptions of Monkeypox versus COVID-19.

The study was approved by the research comity of King Saud University and your participation is voluntary, we aim of this study is to assess the factors affecting parents' acceptance and perceptions of the COVID-19 or Monkeypox vaccine.

The study was approved by the research committee of King Saud University and your participation is voluntary.

Optional: You can provide your email at the end to join the lucky draw for online coupons

For more information, you can email the principal investigator: Emerging Infectious Disease Research Consortium
med.researcher.2020@gmail.com

* 1. (مدى التزام الأسرة مع توصيات الاحترازية لكوفيد (مثل ارتداء الكمامة و التباعد الاجتماعي و تجنب الأماكن المزدحمة
How is the family’s commitment to COVID precautions recommendations (like wearing masks and social distancing and avoiding crowds)

Rarely committed ملتزم نادراً	Medium commitment التزام متوسط	Always committed ملتزم دائماً
☆	☆	☆

* 2. Ageالعمر

- 18-24
- 25-34
- 35-44
- 45-54
- 55-64
- 65+

* 3. Parent’s education: المستوى التعليمي :

- Primary school ابتدائي
- Middle school متوسط
- Highschool ثانوي
- University جامعي
- Master/PhD دراسات عليا (ماجستير - دكتوراه)

* 4. Job-status العمل؟

- Freelance اعمال حرة
- Student طالب - طالبة
- Healthcare worker قطاع صحي
- Employee موظف - موظفة
- Teacher مدرس - مدرسة
- Unemployed لا اعمل حاليا

* 5. هل اصبت انت بكوفيد سابقا؟
Were you affected by COVID-19 yourself?

- No لا
- Yes, but did not require hospitalization نعم لكن لم احتاج التنويم بالمستشفى
- Yes, and required hospitalization نعم و احتجت التنويم بالمستشفى
- Yes, and required hospitalization and ICU نعم و احتجت التنويم بالمستشفى في العناية المركزة

* 6. مقارنة مع الكوفيد: ما هو مستوى القلق لديك حالياً من جدري القرود؟
Compared to COVID-19: how much are you worried nowadays about the Monkeypox?

- Much worried with the COVID-19 قلق اكثر بسبب الكوفيد
- More worried with Monkeypox قلق أكثر من جدري القرود

* 7. هل تعتقد ان مرض جدري القرود مرض خطيرو سريع الانتقال يستدعي اتخاذ إجراءات احترازية تنفسية و تلامسيه؟
Do you think that Monkeypox is a dangerous and rapidly transmitted disease that calls for respiratory and contact precautionary measures to be taken?

- Yes نعم
- No لا

* 8. Smallpox هل تعتقد ان لقاح مرض الجدري
الذي كان يعطى قبل سنة 1980 فعال ضد جدري القرود؟

Do you think that the Smallpox vaccine that was given before 1980 is effective against Monkeypox?

- ◯ Yes نعم
- ◯ No لا
- ◯ I do not know لا أعلم

* 9. هل تعتقد أن لقاح جدري الماء (العنجز) الذي يعطى للأطفال منذ عام 2000 فعال ضد جدري القرود؟

Do you think that the chickenpox vaccine given to children since 2000 is effective against Monkeypox?

- ◯ Yes نعم
- ◯ No لا
- ◯ I do not know لا أعلم

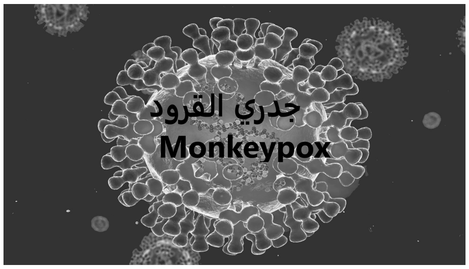

تقييم معلومات البالغين عن جدري القرود مقارنة بالكوفيد

* 10. أسباب القلق من جدري القرود
(الرجاء اختيار كل ما ينطبق)

Worry reasons for the Monkeypox
(Please choose all that apply)

- ☐ me or my family being affected by the Monkeypox disease ان اصاب أنا او احد افراد اسرتي بمرض جدري القرود
- ☐ another worldwide pandemic ان يؤدي المرض الى جائحة ثانية
- ☐ worried Monkeypox might surge to cause national lockdown الزيادة المتسارعة في حالات جدري القرود قد تؤدي الى اعادة الحجر
- ☐ international flight suspension توقف الرحلات الدولية
- ☐ غير ذلك (يرجى تحديدها)
  Other (please specify)

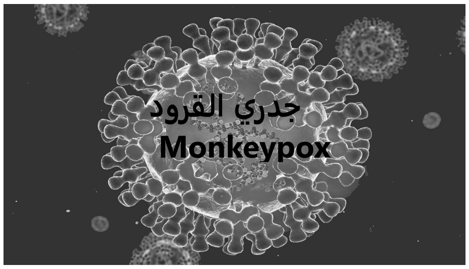

تقييم معلومات البالغين عن جدري القرود مقارنة بالكوفيد

* 11. على حد علمك ، كيف ينتقل جدري القردة إلى البشر؟
To the best of your knowledge, how is Monkeypox transmitted to humans?

- ☐ respiratory secretions الافرازات التنفسية
- ☐ Sexual contact التواصل الجنسي
- ☐ Contact, especially pimples الملامسة خاصة البثور الجلدية
- ☐ Food and drinks الطعام و الشراب
- ☐ Touching contaminated surfaces ملامسة الاسطح الملوثة
- ☐ غير ذلك (يرجى تحديدها)
Other (please specify)

* 12. هل تعلم بان مرض جدري القرود يمكن ان ينتقل قبل ظهور الاعراض خاصة البثور الجلدية؟

Did you know that Monkeypox can be transmitted before symptoms appear, especially skin blisters?

- ◯ Yes نعم
- ◯ No لا

* 13. هل تؤيد التلقيح ضد جدري القرود في هذه المرحلة؟
Do you support the Monkeypox vaccination at this stage?

- ◯ Yes نعم
- ◯ No لا

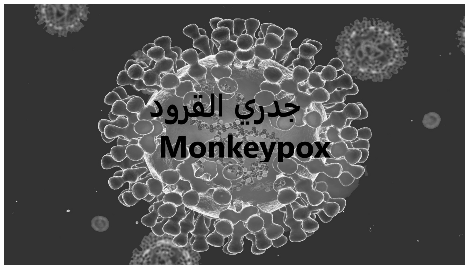

تقييم معلومات البالغين عن جدري القرود مقارنة بالكوفيد

* 14. من الذي يحتاج لتطعيم جدري القرود في رأيك؟
(الرجاء اختيار كل ما ينطبق)
Who do you think needs to be vaccinated for Monkeypox?
(Please choose all that apply)

- ☐ Patients with immune deficiency ضعيفي المناعة
- ☐ Elderly كبار السن
- ☐ Children الأطفال
- ☐ Patients with chronic disease (like DM, hypertension, renal or liver disease, cardiac or chronic lung disease) المصابين بأمراض مزمنة مثل السكري او ارتفاع الضغط او قصور الكلى او تليف الكبد او ضعف عضلة القلب او تليف الرئة
- ☐ Patients with cancer المرضى المصابين بالأورام
- ☐ Healthcare providers الممارسين الصحيين
- ☐ Teachers المدرسين
- ☐ غير ذلك (يرجى التحديد)
Other (please specify)

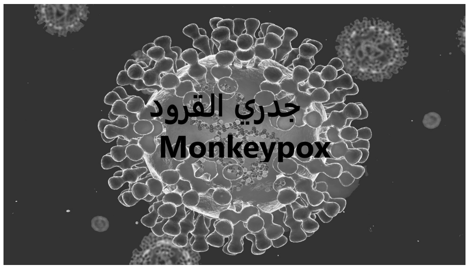

تقييم معلومات البالغين عن جدري القرود مقارنة بالكوفيد

* 15. ما هي مصادر معلوماتك عن جدري القرود بشكل عام؟
(اختر كل ما ينطبق عليك من فضلك)

What are your Monkeypox information sources?
(Please choose all that apply)

- [ ] المواقع الصحية الحكومية المحلية مثل وزارة الصحة - وقاية
  Local government health websites such as the Ministry of Health - Weqaya (Saudi CDC)
- [ ] المواقع الصحية الدولية الرسمية (منظمة الصحة العالمية ومركز السيطرة على الأمراض)
  International health official websites (WHO and CDC)
- [ ] مواقع التواصل الاجتماعي مثل تويتر او الواتساب او الفيسبوك او يوتيوب أو وسائل الاعلام
  Social networking sites such as Twitter, WhatsApp, Facebook, or other media
- [ ] غير ذلك (يرجى التحديد)
  Other (please specify)

16. هل تعلم اذا كان هناك علاج شافي لمرض جدري القرود؟
Do you know if there is a cure for Monkeypox?

- ○ لا يوجد علاج ناجع والعلاج مجرد رعاية عامة
  There is no effective treatment, treatment is just general care
- ○ هناك مضادات فيروسية لعلاج هذا المرض
  There are antivirals to treat this disease

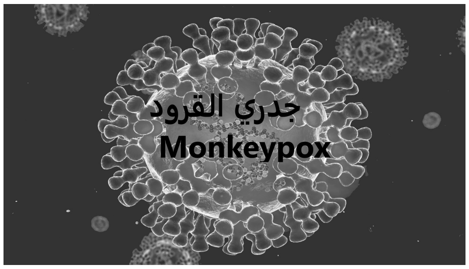

تقييم معلومات البالغين عن جدري القرود مقارنة بالكوفيد

* 17. GAD 7:
Over the last 2 weeks, how often have you been bothered by any of the following problems?
ما مدى تكرار انزعاجك من أي مشكلة من المشكلات التالية خلال الأسبوعين الأخيرين؟

	Not at all أبدا	Several days عدة أيام	More than half the days أكثر من نصف الأيام	Nearly every day كل يوم تقريبا
Feeling nervous, anxious or on edge? الشعور بالتوتر، العصبية أو القلق	○	○	○	○
Not being able to stop or control worrying? عدم القدرة على ايقاف قلقك وهمومك او السيطرة عليها	○	○	○	○
Worrying too much about different things? القلق و الهم الزائد حيال عدة أمور	○	○	○	○
Trouble relaxing? صعوبة في الاسترخاء	○	○	○	○
Being so restless that it is hard to sit still? الشعور بعدم الاستقرار لدرجه تصعب عليك فيها الجلوس بلا حركه	○	○	○	○
Becoming easily annoyed or irritable? الانفعال أو الانزعاج بسهوله	○	○	○	○
Feeling afraid as if something awful might happen? الشعور بالخوف وكأن شيء مريع قد يحدث لك	○	○	○	○

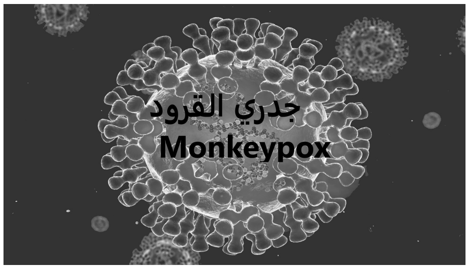

تقييم معلومات البالغين عن جدري القرود مقارنة بالكوفيد

اسئلة اختيارية
Optional questions

18. اختياري: يمكنك كتابة ايميلك للإشتراك في سحب على كوبونات اونلاين
Optional: You can provide your email to join the lucky draw for online coupons

19. كم عدد الأشخاص الذين تعيش معهم؟
How many persons do you live with?

- ◯ 1-3 members
- ◯ 4-6 persons
- ◯ 7-10 persons
- ◯ >=11

* 20. متوسط الدخل الشهري للعائلة:
Mean family’s monthly income

- ◯ Less than 10000 SR أقل من
- ◯ 15000-10000 ريال SR
- ◯ 15001-20000 ريال SR
- ◯ More than 20000 SR أكثر من
- ◯ Prefer not to answer افضل عدم الإجابة

21. هل شجعك هذا الاستبيان على القراءة أكثر عن جدري القرود؟
Did this survey encourage you to read more about Monkeypox?

- ◯ Yes نعم
- ◯ No لا
